# The Immune Response: Targets for the Treatment of Severe Sepsis

**DOI:** 10.1155/2012/697592

**Published:** 2012-12-03

**Authors:** Aline M. Bernard, Gordon R. Bernard

**Affiliations:** ^1^Division of Critical Care, Department of Pediatrics, University of Colorado School of Medicine, 13121 E 17th Avenue, MS 8414, Aurora, CO 80045-2535, USA; ^2^Division of Pediatric Critical Care, Children's Hospital Colorado, 13123 East 16th Avenue, Aurora, CO 80045, USA; ^3^Division of Allergy, Pulmonary and Critical Care Medicine, Department of Medicine, Vanderbilt University School of Medicine, Medical Center North, Vanderbilt University, Room T-1208, Nashville, TN 37232, USA

## Abstract

The clinical process of severe sepsis is characterized by extreme inflammation interlinked with potent stimulation of the coagulation cascade often followed by a state of relative immune paralysis. In this paper, we will review many of the potential therapies directed at various steps along the inflammatory cascade from modulation of inflammatory mediators eliciting the immune response, alteration of the host's immune response in both a stimulatory and depressive manner, and taming the overexuberant coagulation response triggered by the fierce coagulation-inflammation cycle. Finally, we will discuss further opportunities for research to improve our ability to design effective therapies.

## 1. Introduction

The syndrome of severe sepsis is described as a hyperimmune response to one of many infectious insults. It results in an overwhelming surge of cytokines leading to the clinical syndrome of hypotension, multiple organ failure and, sometimes, death [1]. This uncontrolled, hyperimmune response is often accompanied by a state of relative immune paralysis caused by apoptosis of immune cells and high levels of anti-inflammatory cytokines which function to inhibit lymphocytes and macrophages and suppress the production of proinflammatory cytokines. This immune paralysis is postulated to cause the delayed mortality seen in some septic patients due to their inability to oppose and eliminate infections. The balance between hyperimmune response and immune paralysis varies based on patient as well as throughout the course of illness within the same patient [1–3]. 

Sepsis continues to be a significant cause of illness and death worldwide. In the United States alone, it is estimated that it affects more than 750,000 people annually and causes more than 210,000 deaths. Approximately 40% of all intensive care unit patients become septic at some time during the ICU course [3]. 

To date, the sole universally agreed upon treatment for sepsis includes fluids, vasopressors, and source control as defined by the International Surviving Sepsis Campaign Guidelines Committee in 2008. While the therapeutic monitoring goals remain controversial, this strategy of fluid administration and, if needed, vasopressor infusion to restore organ perfusion, source control with a focus on early administration of appropriate broad-spectrum antibiotics, and maximizing oxygen delivery with supplemental oxygen and red blood cell transfusion as indicated is thought to be the most effective approach [4, 5]. Outside of these measures, numerous supplementary strategies have been evaluated without discovery of the perfect antidote.

## 2. Inflammatory Mediators 

Decades ago, unfruitful attempts were made to create antibodies with the potential to bind and to prevent inflammatory bacterial components from triggering the hyperinflammatory response of sepsis. Lipopolysaccharide (LPS), a primary mediator in gram-negative sepsis, was the target of researchers as early as the 1980s. Clinicians tested E5 and HA1A, both anti-LPS monoclonal antibodies, as treatments for septic patients. In initial studies, both antibodies showed encouraging results in small subsets of patients. Fink showed improvement in mortality in patients with culture-proven gram-negative bacteremia when treated with HA1A [6]. Ziegler et al. showed improved mortality with the use of HA-1A therapy in 200 patients with proven gram-negative sepsis. The 343 septic patients without culture proven gram-negative bacteremia showed no treatment benefit [7]. Greenman et al. evaluated E5 in 1991 and showed improved mortality and resolution of organ failure in a subgroup of patients not in shock at the time of study entry [8]. In a follow-up study, Bone et al. evaluated 530 patients with suspected or proven gram-negative sepsis and did not find a difference in mortality but demonstrated improvement of organ failure resolution in those treated with E5 as well as prevention of adult respiratory distress syndrome and central nervous system organ failure [9]. Unfortunately, further studies of these therapies in larger clinical trials including more than 1,000 patients each were unable to confirm efficacy [10–12]. 

More recently, this approach has been revisited with the concept of inhibiting toll-like receptor 4 (TLR-4) which is expressed on the surface of immune cells and binds LPS and other ligands to initiate an intracellular signaling cascade resulting in the release of proinflammatory cytokines [13]. The therapy, TAK-242, functions as a signal inhibitor of the TLR-4 pathway acting after TLR-4 binds with LPS. In septic animal models an improved survival associated with decreased levels of inflammatory cytokines has been shown with the use of this therapy. Furthermore, its use in healthy volunteers prior to instillation of LPS also resulted in decreased levels of inflammatory cytokines when these patients were given an LPS challenge. In 2010, Rice et al. evaluated TAK-242 in a randomized, placebo-controlled trial of patients with severe sepsis and shock or respiratory failure. High-dose and low-dose treatment regiments were compared to placebo with primary endpoints of change in IL-6 level and 28-day mortality rate. This trial was terminated after enrollment of 274 patients failed to show suppression of IL-6 levels. Evaluation of the treated patients showed no difference in 28-day mortality compared to placebo, however, there was a trend toward improved survival in those with both shock and respiratory failure who were in the higher treatment dose cohort [14]. It may be that this therapy could be effective in patients with a higher severity of illness, as suggested by the trend towards improved survival of the patients with both respiratory failure and shock. Furthermore, the mean time from onset of shock or respiratory failure to initiation of TAK-242 therapy was 19 hours. The dynamic nature of the immune response has been well described and it could be postulated that the delay of 19 hours is too long for the treatment to have the ability to suppress the immune response.

## 3. Steroids

Steroids act early in the inflammatory cascade eliciting a wide range of effects via broad suppression of the immune system and are hypothesized to provide benefit as a supplementary treatment of sepsis. Steroids function by inhibiting production of proinflammatory cytokines (TNF-*α*, IL-1, IL-2, IL-6, and IFN-gamma), chemokines, bradykinins, and eicosanoids. Simultaneously, they increase anti-inflammatory mediators (IL-10, IL-1 receptor antagonists, and TNF receptor antagonists), inhibit inducible nitric oxide synthase, decrease migration of inflammatory cells to sites of inflammation, and reduce the function of inflammatory cells [1]. It is further postulated that steroids increase the expression of adrenergic receptors in the vasculature. These receptors are downregulated in septic shock and, theoretically, increasing their expression allows the vasculature to respond to the high levels of circulating cortisol [2].

Initially, studies were done using high-dose steroids (e.g., 30 mg/kg methylprednisolone) with the goal of broad suppression of the body's overreactive inflammatory response [15–17]. These studies failed to show benefit and even showed a trend towards harm including increased mortality due to secondary infections in those treated with steroids, thus, causing steroid therapy use to decrease in the early 1990s [2, 17]. The use of steroids was revived in the mid 1990s with the target of treating relative adrenal insufficiency with the use of replacement, low-dose glucocorticoids. The treatment with low-dose steroids is thought to improve vascular response to endogenous and exogenous catecholamines via the upregulation of adrenergic receptors in the vasculature. While avoiding the substantial immune system blockade, this lower dose is thought to maintain some anti-inflammatory effects via preventing release of proinflammatory cytokines and activation of endothelial cells and neutrophils to decrease sepsis triggered clotting disorders [15, 16]. 

Small studies done in the late 1990s showed trends toward improvement in hypotension and mortality with the low-dose steroid treatment strategy. However, these studies were underpowered to detect clinically significant effects [1]. More recently, two large randomized, controlled trials have been published that further evaluated the effectiveness of steroid therapy. In 2002, a study completed by Annane et al. evaluated 300 patients with septic shock and showed improvement of refractory hypotension and a decrease in absolute mortality in patients with relative adrenal insufficiency treated with 7 days of hydrocortisone and fludrocortisone. This study also showed that adrenal-sufficient patients as defined as displaying a response to ACTH gained no benefit and trended towards harm from glucocorticoids [18]. Subsequently, the CORTICUS study published in 2008 by Sprung et al. compared the treatment of 499 septic patients with 11 days of hydrocortisone versus placebo. Contrary to the Annane study, the CORTICUS trial failed to show an improvement in mortality or reversal of shock in treated patients, regardless of ACTH response. They did, however, show a faster resolution of shock in those patients who had shock resolution and were treated with hydrocortisone. Interestingly, they did show an increased incidence of superinfection in those treated with steroids [19]. The differences in the outcomes of these trials may be linked to several key differences between them. The populations studied included different timing of patient enrollment. The Annane study took patients up to 8 hours after onset of shock while the CORTICUS study extended their enrollment up to 72 hours after the onset of shock. Furthermore, the CORTICUS trial included all patients in shock while the Annane study restricted their study to only those who were both fluid and vasopressor refractory. Similarly, the patients in the Annane study were significantly more ill at baseline with a higher SAPS score and a higher mortality rate in the placebo groups than the CORTICUS trial (65% Annane trial versus 32% CORTICUS trial). It could be postulated that their conflicting results are a product of their differing patient populations as the Annane study evaluated a group of patients with a higher degree of illness and more refractory shock [4, 16].

Furthermore, the Annane and CORTICUS trials differed regarding the utility of the ACTH stimulation test. The Annane study showed that ACTH nonresponders were more likely to benefit from steroid therapy while the CORTICUS trial failed to replicate this finding [18, 19]. Given the challenges of measuring cortisol levels and the finding that the Annane trial showed an overall trend toward benefit of steroid therapy, regardless of ACTH responsiveness, the 2008 Surviving Sepsis Guidelines recommended that the ACTH stimulation test not to be used as a tool to guide the use of steroid therapy [4]. Known side effects of steroids including hyperglycemia, gastrointestinal bleeding, myopathy, and secondary infection have tempered the enthusiasm for steroid use. The CORTICUS trial, showing no efficacy of steroids reinforced these reservations when it also demonstrated an increase in episodes of superinfection with new sepsis and septic shock in those treated with steroids [16, 19]. 

Currently, the adult literature has not developed a standard of care in regards to steroid therapy. The Surviving Sepsis Guidelines recommend the use of steroids only in fluid and vasopressor refractory shock and do not recommend the use of the ACTH stimulation test based on low-grade and moderate-grade evidence, respectively [4]. Furthermore, they advise tapering the steroids when the state of shock resolves. Less data exists in regards to the pediatric population and the Surviving Sepsis Guidelines base recommendations on a retrospective review done by Markovitz et al. in 2005 [20]. This review showed that corticosteroid use in children with severe sepsis was an independent predictor of mortality. However, the nature of the study design does not allow for causal inference and the Surviving Sepsis Guidelines cite a weak recommendation based on low-grade evidence for the use of hydrocortisone only in children with catecholamine resistant shock and suspected or proven adrenal insufficiency [4]. Ideally, what is needed is a better means of determining the population of septic patients which have the best chance to benefit from steroid treatment while having the least risk of harm due to the side effects of the therapy.

## 4. Antagonism of Proinflammatory Cytokines

Knowledge of the inflammatory cascade and, more specifically, proinflammatory cytokines has allowed specific targets for immunosuppression including TNF-*α* and IL-1. TNF-*α* injection into animals has been shown to trigger a sepsis-like syndrome including hypotension, activation of the clotting cascade, significant organ dysfunction, and even death. Furthermore, increasing and persistently elevated levels of TNF-*α* are associated with nonsurvival in humans [21, 22]. Downstream effects of TNF-*α* include augmentation of the inflammatory cascade via elevation of multiple cytokine levels and upregulation of adhesion molecules on leukocytes, platelets, and endothelial cells. TNF-*α* also stimulates the coagulation system via activation of thrombotic and fibrinolytic pathways. Despite the deleterious effects of this overstimulation, it is evident that TNF-*α* plays a crucial role in the immune system because blockage of its activity in animal models has led to a worsened ability of the animal's immune system to clear microbes [21]. Due to its pivotal position in the inflammatory and coagulation systems that are known to cause the demise in sepsis, TNF-*α* has been targeted as a treatment of sepsis in many clinical trials. Although no trial has succeeded in showing an overall improvement using this therapy, several studies have identified populations and/or characteristics of these patients that may direct future trials.

The first large trial, NORASEPT, was done by Abraham et al. in 1995 and included 900 patients with sepsis or septic shock. The NORASEPT trial evaluated an anti-TNF-*α* monoclonal antibody and failed to show an overall mortality benefit. However, the subset of patients with septic shock showed a significant improvement in mortality 3 days after drug infusion. In following the patients further, the 28-day mortality continued to show a trend towards improvement but was no longer significant [23]. The INTERSEPT study, published in 1996, focused on evaluation of 420 patients with septic shock. This study showed more rapid reversal of shock and fewer patients with at least one organ failure in survivors who were treated with the anti-TNF-*α* monoclonal antibody as compared with the placebo group. However, this trial failed to show a difference in mortality [24]. This drug was tested in a third trial, NORASEPT II, which also failed to show an improvement in mortality [25]. 

A trial of an anti-TNF-*α* antibody fragment, afelimomab, was done by Reinhart et al. and published in 1996 that suggested a benefit of treatment in patients with baseline elevation of IL-6 [26]. Physiologically, this association is plausible as IL-6 levels are considered to be a surrogate for overall TNF-*α* activity due to the longer half-life of IL-6 compared to the rapidly cleared TNF-*α*. This hypothesis was tested in a prospective, randomized placebo-controlled trial, the RAMSES study of 446 patients with elevated IL-6 levels. It showed a nonsignificant trend towards improved survival in those treated with afelimomab [27]. A later study, the MONARCS trial, tested the same antibody fragment in 998 patients with elevated IL-6 levels and found a trend towards improved survival in treated patients as compared to placebo. The risk-adjusted reduction in mortality was 5.8% and corresponded to a relative risk reduction for mortality of 11.9%. This study also found a greater reduction in IL-6 levels and multiorgan dysfunction score in those treated with afelimomab. The results are also encouraging because patients with higher IL-6 levels had significantly higher mortality rates in the placebo group than those with lower IL-6 levels. Thus, this showed that afelimomab had a greater effect in patients at higher risk of mortality [28]. In a similar investigation, cytofab, a preparation of polyclonal ovine anti-TNF Fab IgG fragments, was tested in a phase II placebo-controlled randomized clinical trial in 81 septic patients with shock or two organ dysfunctions. While this study did not show a difference in mortality, the investigators were able to show an increase in ventilator-free days, ICU-free days, and a decrease in serum and BAL levels of TNF-*α* and downstream effects on IL-6 in patients treated with CytoFab [29].

The persistent trends toward improved survival in the above studies are encouraging that some patients have the ability to benefit from immunotherapies. The difficulty lies in determining which patients are most likely to benefit. Are elevated IL-6 levels the correct marker for the selection of patients for use of anti-TNF-*α* therapy? Are elevated IL-6 levels a marker of worsening disease severity and, thus, improvement in this group of patients is due to their high severity of illness at presentation? Are IL-6 levels a reflection of timing of progression of sepsis? It is the hope that with further research, clinicians will be able to determine exactly which target population and at what point in their disease patients will benefit from a given treatment such as anti-TNF-*α* therapy. 

With a similar mechanism of action, IL-1 is also a target of immunotherapies. This proinflammatory cytokine works together with TNF-*α* to propagate the hyperimmune response of sepsis. Macrophages and other cells naturally produce IL-1 receptor antagonist (IL-1ra) in response to IL-1, endotoxin, and various other microbial elements. The IL-1ra reversibly binds and competitively inhibits IL-1 receptors [30]. In 1994, Fisher et al. published a study evaluating the use of IL-1ra in the treatment of 893 patients with sepsis. This study failed to show an overall increase in survival in those treated as compared to placebo. However, retrospective and secondary analyses identified a trend of increased survival among patients with sepsis as well as an organ dysfunction and/or a predicted risk of mortality ≥ 24% [30]. Subsequently, Opal et al. published a trial in 1997 focusing on IL-1ra treatment in patients with severe sepsis and/or septic shock. Disappointingly, this study was halted when just over half of the proposed enrollment was completed and analysis revealed a low likelihood of showing a statistical difference in their primary endpoint, 28-day mortality. Secondary endpoints showed that those patients treated with IL-1ra displayed a nonsignificant trend towards improvement of organ dysfunction. The authors postulate that they may have had greater success if they were able to identify a more homogenous population. They were also concerned that their treatment was unable to maintain the necessary 100–10,000 fold excess of IL-1ra relative to IL-1 as it is known that stimulation of as few as 5% of the IL-1 receptors triggers an inflammatory response [31]. Perhaps further evaluation of this drug with the monitoring of levels to ensure complete blockage of the receptors or use of the drug in a more targeted population would provide a better chance for success.

## 5. Statins

There are many ways that statins have the ability to affect the immune response in sepsis and the exact mechanism of their action is unknown. Statins inhibit the reduction of hydroxymethyl-glutaryl-CoA to mevalonate which plays a role in synthesis of bile acids, some steroid hormones, and vitamin D. Statins inhibit various other pathways involved in pathophysiology of sepsis including inhibition of the production of cyclo-oxygenase-2 protein, biosynthesis of ubiquinone which functions in the electron transport chain of mitochondria, heme-A used in oxygen transport, and prenylation of small G proteins. It is likely that the alteration of the G-protein pathways has the most influential effect as this significantly alters inflammatory cell activation and protein production. Among other proteins, it is known to inhibit the production of subunits necessary for the GTP binding protein Rho. This inhibition has the downstream effect of production of a decreased amount of inflammatory cytokines such as IL-6 and IL-1. Furthermore, HMGCoA-reductase also induces caspase-dependent apoptosis in smooth muscle cells that may result in less inflammation due to avoidance of necrotic cell death [32].

Data from prospective, randomized-controlled trials evaluating the use of statin therapy in sepsis is lacking. However, multiple observational studies show encouraging effects. A large cohort study of more than 12,000 critically ill patients was published by Christensen et al. in 2010. Results showed that patients on statin therapy immediately prior to ICU admission had a decreased risk of mortality within 30 days and up to 1 year after ICU admission. Given the design of this study, the authors are unable to infer causation but the results stimulate excitement for further evaluation of the effects of statin use [33]. A large meta-analysis done by Bjorkhem-Bergman et al, published in 2010, evaluated the potential use of statin therapy in bacterial infection. It showed that patients on statin therapy seemed to have better outcomes including decreased mortality. However, when the 15 observational studies were adjusted for publication bias the association failed to reach statistical significance [34]. During that same year, Janda et al. focused the evaluation further when they published a meta-analysis evaluating statin therapy in severe infections and sepsis. This study included 20 trials, mostly cohort studies and one randomized-controlled trial that demonstrated a protective effect associated with statin use. The positive outcomes evaluated included 30-day mortality, in-hospital mortality, pneumonia-related mortality, bacteremia-related mortality, sepsis-related mortality, and mixed infection related morality. Again, this study was limited due to the inclusion of mostly cohort studies and significant heterogeneity of trials [35]. The one randomized controlled trial in this data set was completed by Tseng et al. and included 80 patients with aneurysmal subarachnoid hemorrhages. While this study did show an improvement in sepsis-associated mortality, it cautioned that this finding was a secondary outcome [36]. Due to the promising effects of statins, both based on physiologic knowledge and on the current observational data, phase II and phase III studies are currently in progress to evaluate the role of statins in the treatment of sepsis.

## 6. Inhibition of the Coagulation Cascade

The extreme activation of the inflammatory system in severe sepsis is accompanied by a potentially equal stimulation of the coagulation system. From an adaptive perspective, this interaction is logical as the activation of the coagulation system can be envisioned as an effort to isolate the infection with the goal of limiting its spread throughout the body. However, in the process of severe sepsis, this activation results in a futile and, likely, counterproductive endeavor as the infection has already spread throughout the bloodstream and the coagulation system activation results in diffuse microvascular thrombi with wide spread endothelial damage and organ failure. 

Various steps of the coagulation pathway have been targeted in the treatment of sepsis. Tissue factor (TF), a cell surface receptor whose expression by endothelial cells and monocytes occurs in the presence of inflammatory mediators, acts to initiate the extrinsic coagulation pathway. A TF inhibitor was tested in the Phase III trial, OPTIMIST, evaluating its use in 1,754 patients with severe sepsis and this trial failed to show an improvement in mortality. More concerning, it showed a trend towards harm in those treated concurrently with heparin [37]. Similarly, antithrombin III (AT III), an anticoagulant, was the subject of sepsis therapy as well due to the finding of decreased AT III levels in severe sepsis and the hypothesis that this deficiency contributes to the hypercoagulation pathophysiology in sepsis. Multiple small studies published in the 1990s showed promising results. However, in a phase III trial of 2,314 septic patients, they were unable to show a difference in overall mortality. However, in subgroup analysis, patients not treated concomitantly with heparin showed a significant decrease in mortality at 90 days while those treated with heparin showed a significantly increased risk of bleeding [38]. Future investigation of AT III as a treatment for sepsis will need to carefully select their target population to ensure minimal risks for bleeding.

To date, the only drug that has been approved for the treatment of severe sepsis is recombinant human activated protein C (rhaPC). It was investigated due to its anti-apoptotic, anti-inflammatory, and anticoagulant effects. It acts via inhibition of factors Va and VIIIa which results in the prevention of thrombin generation. Downstream, this decreases inflammation by reducing mast cell degranulation, platelet activation, and neutrophil recruitment [5, 39]. The PROWESS trial, published in 2001 spurred great excitement due to its absolute reduction in 28-day mortality by 6.1% in septic patients treated with rhaPC and it was subsequently approved for use in the most severely ill septic patients with APACHE scores greater than 25 as this subgroup seemed to derive the most benefit from treatment [39]. Unfortunately, these results were not replicated in the PROWESS-SHOCK study and the treatment was voluntarily removed from the market by the manufacturer [40]. The use of rhaPC is not recommended for use in children based on a study published in 2007 that evaluated 477 septic children and failed to show an improvement in mortality [41].

Thrombomodulin (TM), another naturally occurring pathway in the coagulation system, is currently being targeted in the treatment of sepsis. TM, produced by endothelial cells, acts upstream in the activated protein C pathway to sensitize the thrombin receptor leading to activation of protein C [42]. It has been shown that the serum concentration of TM parallels the severity of coagulopathy and organ failure in sepsis and decreases as DIC and ARDS improves [43]. A control study of 20 patients with severe sepsis-induced DIC treated with rhTM compared to 45 historical controls showed improved 28-day mortality and improved organ dysfunction in those treated with rhTM [42]. Ongoing phase II studies are in progress to evaluate the efficacy of rhTM [5].

## 7. Immunostimulation

Due to the recognition that sepsis is characterized by a combination of hyperimmune response and relative immunoparalysis, further investigations have pursued immunostimulatory strategies. A controversial and widely studied therapy is treatment with the use of pooled serum polyclonal immunoglobulin preparations, IVIG. Although the exact mechanism remains in question, it is thought that the immunoglobulins coat bacteria, which improves phagocytosis and enhances neutralization and opsonization causing inactivation of bacterial endotoxins and exotoxins. Furthermore, it is hypothesized that the treatment alters the release of cytokines and cytokine antagonists by endotoxin and interacts with the complement cascade causing an improved immune response in sepsis [10]. Further supporting this strategy is a recent study which evaluated 62 adult septic patients and revealed decreased levels of immunoglobulins particularly IgG and IgM early in sepsis as compared to age-matched controls. This was followed by normalization of levels after 7 days in the majority of patients. Decreased level of immunoglobulins was associated with decreased levels of plasma proteins but was not associated with a difference in mortality [44]. 

In 2007 and 2008, three meta-analyses were published that evaluated the efficacy of polyclonal IVIG in adult patients with sepsis. All three concluded that this therapy improved survival but, due to small study sizes, heterogeneity, and methodologic limitations of the individual studies, the three authors recommended large randomized, controlled trials to verify therapeutic efficacy [45–47]. A subsequent Cochrane review published in 2010 evaluated 17 trials of polyclonal IVIG in adult patients with sepsis. This review was in agreement with the prior meta-analyses and showed a reduction in less than 30-day mortality in treated patients. However, the authors recommended cautious interpretation of their findings as the majority of studies had a small sample size and there was concern for poor methodologic quality. Furthermore, when the trials were restricted to those with low risk of bias, no reduction in mortality was shown and the studies that evaluated long-term mortality (greater than 60 days) did not show an effect. The Cochrane review went on to specify their agreement with the Kreymann et al. meta-analysis findings that the IgM-enriched formulation of immunoglobulin is also beneficial and even trended toward a greater effectiveness in the treatment of sepsis [10, 45]. The authors conclude that polyclonal immunoglobulins appear to be beneficial as adjuvant therapy for sepsis but recommend large, multicenter studies for confirmation [10].

The pediatric population stands to reap greater benefit from IVIG due to the immaturity of B-cells in patients less than 5 years old. In 2005, a prospective case-controlled trial of 100 pediatric patients showed a significant improvement in length of stay, development of complications, and mortality in septic pediatric patients 1 month–24 months old treated with IVIG [48]. Based on the findings of this study, the Surviving Sepsis Guidelines recommend consideration of IVIG treatment of pediatric patients with severe sepsis. However, this recommendation is supported only by weak evidence due to low trial quality [4]. IVIG in the neonatal population is equally as controversial as the Cochrane review found no reduction in mortality in septic neonates treated with IVIG while the Surviving Sepsis Guidelines cite that there is evidence to support improved mortality in neonates treated with IVIG [4, 10]. A study published by Brocklehurst et al. in 2011, after the publication of the Surviving Sepsis Guidelines and Alejandria's Cochrane review, evaluated over 3,000 neonates with sepsis and found no difference in the primary outcomes of mortality or major disability up to two years of age [49].

Other immunostimulatory strategies include cytokine stimulation with granulocyte-colony stimulating factor (G-CSF), granulocyte-macrophage colony stimulating factor (GM-CSF), and IFN-gamma. The hypothesized mechanism of these therapies in nonneutropenic patients is stimulation of bactericidal activity via increased leukocytosis and increased activity of granulocytes. Bo et al. published a meta-analysis of 21 randomized-controlled trials evaluating G-CSF and GM-CSF in the treatment of sepsis. This evaluation of a combined 2,380 septic patients showed no change in mortality but did show a positive effect of this therapy on the rate of reversal of infection. They found no difference in adverse events between the groups and recommended further studies to evaluate the efficacy of this therapy [50]. It is important to remember that G-CSF and GM-CSF differ in that GM-CSF has additional monocytic and macrocytic stimulatory affects, inducing monocytic cytokine expression and antigen presentation via increased expression mHLA-DR theoretically resulting in improved adaptive immunity [51]. In the meta-analysis by Bo et al., the data evaluating these two therapies were combined despite their differing mechanisms of action. Furthermore, the studies differed significantly on dose as well as on the route of administration and failed to stratify the patients based on their immunologic state. Given the immune-stimulatory mechanism of this therapy, it would be important to know if the patients being studied are in the hyper or hypoimmune phase of sepsis as this may affect the drug's efficacy. Furthermore, new data has shown that it is possible to track the efficacy of immunostimulatory therapies by measurement of mHLA-DR expression on monocytes which is decreased in patients with sepsis-associated immune cell dysfunction [51, 52]. Meisel et al. recently showed that patients with sepsis-associated immunosuppression, defined as low monocytic HLA-DR expression, who were treated with GM-CSF had improvement of monocytic HLA-DR expression when compared to placebo patients. Although this trial included only 38 patients, they were able to show shorter duration of mechanical ventilation as well as shorter ICU and hospital lengths of stay [52].

IFN-gamma has shown similar ability to restore monocytic HLA-DR expression in septic patients with evidence of monocyte deactivation. A small study done by Docke et al. showed that IFN-gamma treatment in septic patients with low monocytic HLA-DR expression resulted in restoration of monocyte function as measured by improved TNF-*α* secretion resulting in clearance of sepsis in 8 of 9 patients [53]. The results of these studies are encouraging that immunostimulation may be an effective way to treat the subset of septic patients who are in the immunoparalysis phase of their disease.

## 8. Directions for Future Research

Given the incredible number of patients affected with and dying due to sepsis, it is disappointing that the proven treatments of this disease have not expanded beyond that of fluids, vasopressors, and source control. To date, the knowledge gained form laboratory research and clinical trials has better defined the pathophysiology of the disease and the population of patients we are treating. However, rather than clearly providing new treatments, it has left us with more questions than answers. Despite the disappointing results of clinical trials which have been unable to find a universal treatment effective in all patients, some of the trials have shown promise in specific groups of patients. For example, HA-1A may improve outcomes in patients with gram-negative infections and anti-TNF-*α* therapies may effectively treat patients with elevated IL-6 levels despite these individual therapies' inability to treat all-comers with sepsis. TLR-4 inhibitors such as TAK-242 may have greater effect when used earlier in the course of the illness or when used in the most severely ill cohort of patients. Immunostimulatory medications may improve patients in the hypoimmune phase of illness as identified by low monocytic HLA-DR expression or another yet-to-be identified marker of the immunoparalysis phase. Therapies with more hazardous side effect profiles such as steroids or activated protein C may prove to be efficacious in the most severely ill patients or those with greater coagulopathy, where the risks of the disease progression outweigh the risks of the therapy. Even better, we may develop a test or clinical profile that will allow us to better identify the patients most likely to benefit from the specific therapy or provide the subset of patients at minimal risk of an adverse event. It is also conceivable that concurrent therapy will help dictate the best treatment option. For example, a study of AT III restricted to patients without concomitant anticoagulation therapy may show that it can serve as a beneficial treatment for sepsis.

Further research may help to clarify the role of genetics in improving individualization of therapies as well. For more than ten years, genetic studies have evaluated links between polymorphisms of the major histocompatibility complex genes on chromosome 6 and human leukocyte antigen genes to the body's response to infection [54]. While it has yet to translate into clinically significant data, genetic studies have identified various polymorphisms associated with an increased risk of infection. Many of these polymorphisms cause alterations in the body's immune response. For example, one such polymorphism lies in the promoter region of TNF-*α*. A second polymorphism causes alterations of the two well-studied toll-like receptors, TLR2 and TLR4, which provide the innate immune system with the ability to recognize and respond to gram-positive and gram-negative bacteria. Further studies have evaluated expression profiling via measurement of mRNA. These studies and the help of computer technology have led to identification of subclasses of children with septic shock based on similar patterns of gene expression [55]. With an improved ability to link genetics to a patient's specific disease process and, further, to therapeutic response, a more customized approach to therapy could be achieved both by directing specific therapies as well as by creating more homogenous populations of study patients with an improved ability to show efficacy in clinical trials. To add a greater degree of complexity, perhaps the use of multiple therapies in order to attack the disease process from different approaches will prove to be the best customization of therapy.

It is the above variables including but not limited to the infecting organism, phase of illness, severity of illness, host's inflammatory response, and genotype that make this disease process exceedingly difficult to combat. Ideally, it would be possible to construct homogenous septic patient populations in order to appropriately test various therapies in subgroups of patients. However, no single ICU has the ability to generate the numbers necessary in order to evaluate and prove efficacy of these personalized treatment strategies. On the other hand, modern computer technology allows us to search and evaluate large sums of data. Not only does this give us the opportunity to pool data from multiple sites in an efficient manner but also it allows us to search this data in a very sophisticated way. In doing so, it may be possible to identify patterns based on clinical symptoms, laboratory studies, comorbidities, genomic information, response to therapy, biomarkers, and so forth, that will enable us to form groupings of patients and monitor their response to treatment options carefully selected based on our knowledge of the mechanism of action of the therapy and understanding of the patient's disease process.

In 2004, Science Applications International Corporation and Merck Capital Ventures studied the advances in technology and the factors influencing their adoption rates in the use of clinical trial development. The study gathered information by reviewing industry-sponsored research, performing a literature research and interviewing those with significant experience in clinical development process, especially in business processes and information technology (IT). Their study showed a significant resistance of moving away from paper-based system and to new IT. In contrast, it also showed an increasing acceptance of IT in the face of regulatory pressures to improve adverse event reporting and improve the success of submissions. Furthermore, they found that process change is the key to improving the core function of the system and that the addition of technology alone, without alteration of existing processes, is not sufficient. Their belief is that IT can benefit clinical research in the ways of improved cycle time, data quality, and cost effectiveness. Current clinical trial structures are fraught with incompatible systems, complicated data entry formats and challenging organization for data searching as well as exchanging of information. This study sites outcomes such as centralization of data, advanced data mining capabilities, vocabulary standards, and cross-trial data pooling that could be achieved by adoption of new IT pathways to advance the field of clinical trials [56]. In 2004, the FDA presented the report, *Innovation or Stagnation, Challenge and Opportunity on the Critical Path to New Medical Products*, which detailed its concerns regarding the field of drug development. It described a 50% decline in new product submissions to the FDA over the previous 10 years in spite of a 250% increase in research and development expenditures. Their analysis found that 50% of drugs that showed promise in phase II trials went on to fail in phase III studies and that only one in ten drugs that undergo clinical testing eventually obtain FDA approval. Furthermore, it takes an average of 15 years and nearly a billion dollars in research and development to reach the clinical market. They cited the major component of the inefficiency in drug development as the lack of modern methods for drug testing stating, “Often, developers are forced to use the tools of the last century to evaluate this century's advances.” [57]. The efforts to improve the field of clinical research to the level of technology incorporated in other areas of business will be well worth the investment. The outcomes will benefit the patients we care for by providing an improved understanding of the methods to combat sepsis and the ability to deliver of the most up-to-date treatments.
